# Journeys, Journey Conditions, and Welfare Assessment of Unbroken (Unhandled) Horses on Arrival at a Slaughterhouse in Italy

**DOI:** 10.3390/ani12162083

**Published:** 2022-08-15

**Authors:** Martina Zappaterra, Leonardo Nanni Costa, Martina Felici, Michela Minero, Francesco Perniola, Daniele Tullio, Barbara Padalino

**Affiliations:** 1 Department of Agricultural and Food Sciences, Alma Mater Studiorum—University of Bologna, 40127 Bologna, Italy; 2 Department of Veterinary Medicine, University of Milan, 20122 Milano, Italy; 3 Veterinary Clinic ‘La Fenice’, 70029 Santeramo in Colle, Italy; 4 ASL BA—Local Health Authority Veterinary Service, Via dei Mille 29, 70126 Bari, Italy

**Keywords:** ABMs, EBMs, horses, transport stress, journey conditions, welfare

## Abstract

**Simple Summary:**

In Europe, many horses travel long distances to reach a slaughterhouse and it is claimed that many of them are unbroken and travel in poor conditions. Hypothesizing that journey conditions would be crucial to protecting the welfare of unbroken horses, this study aimed to describe their journeys and journey conditions, document their welfare status on arrival at a slaughterhouse, and investigate possible associations between journey conditions and welfare issues. A protocol to assess the welfare of the transported horses at unloading and during lairage was developed and applied on a total of 395 unbroken draft horses from 20 different consignments at a slaughterhouse in Southern Italy. The average journey duration was 34 h, coming from France and Poland. Trucks were well equipped and driven by experienced staff, while horses traveled loose and in small groups. At arrival, the horses self-unloaded and the prevalence of health problems was minimal (1.52% injuries; 4.30% nasal discharge; 6.58% abnormal feces). Space allowance, lack of feeding during transport, and cold temperatures were determined to be the main risk factors for their health and welfare. When unbroken horses travel over a long distance, the way in which they are transported (i.e., journey conditions) is crucial and must be maintained at a high standard to minimize the risk of the animals’ welfare becoming compromised.

**Abstract:**

Transportation of horses to slaughterhouses can pose a welfare concern, in particular when horses are unbroken/unhandled. This study aimed to describe their journeys and journey conditions, document their welfare status on arrival in a slaughterhouse in Italy, and investigate possible associations between journey conditions and welfare issues. A total of 395 unbroken draft horses in 20 different consignments were assessed with a standardized protocol. The most common departure point (16/20 consignments, 80%) was a French assembly center, but many of these horses had Spanish passports, suggesting they had previously been transported from Spain to France. The average journey duration was 34 ± 14 h, including journey breaks (i.e., short stops inside the vehicle and long resting stops at control posts), while transit time was 24 ± 4 h. The drivers were well experienced, the trucks were well equipped (i.e., forced ventilation, drinkers), and the horses traveled loose in small groups (*n* ≤ 4 horses) within pens inside the vehicle. On arrival at the slaughterhouse, the horses self-unloaded and showed minimal behavioral and health problems. The prevalence of injuries, diarrhea/abnormal feces, and nasal discharge were 1.52%, 6.58%, and 4.30%, respectively. Cold temperatures, space allowance, and lack of feeding during transport were found to be the main hazards of those problems (all *p* < 0.05). Our findings confirm that the correct assessment of the fitness for transport, adequate journey conditions, and experienced staff are crucial factors to safeguard the welfare of unbroken horses during long journeys.

## 1. Introduction

The commercial transportation of horses to slaughterhouses is a welfare concern [1]. Globally, the number of horses slaughtered for human consumption has increased in recent years [2], and horses are usually slaughtered in Asia, South America, and Europe after long and short journeys [3,4]. Within Europe, Italy, France, Belgium, and Holland are among the major producers or consumers of horsemeat, but horses are primarily sourced from Romania, Poland, Spain, Bulgaria, Lithuania, Hungary, and Belarus [1,5]. Even countries where horses are not kept for meat contribute to the horsemeat trade by shipping horses at the end of their career to countries where they can be slaughtered [6]. Therefore, the distance between countries of origin and slaughter creates a trade involving large numbers of live horses being transported over long distances. Moreover, it is claimed that the majority of those horses are unbroken and are transported in poor conditions [6,7].

The evidence of the effects of transportation to slaughter is scant [8]. While dead animals on arrival (DOA) or in the lairage were never recorded, the prevalence of severe welfare issues varied from study to study [1,9,10,11]. Marlin et al. [1] found that 23% out of 1519 horses unloaded in Italy after long journeys (arriving from Poland and Romania) had at least one acute injury and 10% were severely lame. Messori et al. [9] reported that only 0.1% of the considered 926 horses unloaded in Italy (arriving from Poland) showed severe lameness, but no horses were unable to move or were severely injured. Roy et al. [12] reported that out of 3940 horses transported to Canadian slaughterhouses, only 1% of them were lame, and unable to walk in 0.16% of cases. Finally, Miranda-de la Lama et al. [11], concerning a total of 2648 horses transported to Mexican slaughterhouses, reported that 0.75% of horses from the USA and 0.30% of horses from Mexico were non-ambulatory at arrival and that 18.6% of them showed a “very poor” profile based on the welfare indicators such as lameness, nasal discharge, ocular discharge, and skin wounds. Since there is no official and validated method to assess the welfare of horses during and after traveling, it is difficult to compare the conditions and outcomes [8].

Several hazards linked to transport-related welfare issues have been identified [8]. Improper assessment of the fitness for transport, lack or insufficient provision of water and food in transit, high stocking density, journey duration, and high effective temperatures inside the vehicles were all considered important risk factors [11,12,13]. However, Miranda-de la Lama et al. [11] showed that there were no differences between the welfare status of horses arriving from Mexico and the USA, despite different journey durations. Moreover, over the years, there seemed to be a reduction of the health and welfare problems, mainly due to a mitigation of hazards, and, consequently, an improvement of the journey conditions under which horses were transported to the slaughterhouse. This improvement may be due to increased compliance and better enforcement of the codes of transport worldwide.

In Europe, Council Regulation (CE) 1/2005 [14] sets out the legal journey conditions that must be met, depending on the species and categories of the transported animals. For horses, it allows journeys over long distances (more than 8 h) to be only undertaken by broken (handled) horses, while for unbroken (unhandled) horses the maximum journey duration is set at 8 h. Unbroken horses shall also travel loose and in small groups of familiar horses (*n* ≤ 4 horses). These requirements are based on the evidence that unbroken horses struggle to cope with new situations and being tied or isolated [9,15,16,17]. Despite these different requirements for the journey conditions, in the current regulation, it is not clear how to differentiate between broken and unbroken horses. For this reason, Menchetti et al. [18] validated a simple and fast behavioral test that allows an individual horse’s level of “tameness” to be identified, and suggested that this test should always be carried out before loading to ensure journey conditions are adapted accordingly. Hypothesizing that journey conditions (e.g., space allowance, driving style and driver’s experience, environmental conditions) would be crucial for the welfare of unbroken horses, this study aimed to describe their journeys and journey conditions towards a slaughterhouse in Italy, document their welfare status at unloading and lairage using a standardized protocol, and investigate possible associations between journey conditions and welfare issues.

## 2. Materials and Methods

### 2.1. Sample Size Calculation

In 2021, 29,937 horses were slaughtered in Italy, 30.75% of which (about 9206) were slaughtered in Apulia, a region of Southern Italy [19]. A power analysis was therefore conducted using Statulator^®^ [20] to determine the sample size to include in a 1-year cross-sectional study for a target population estimated at 9206 horses. The number of animals to be assessed was estimated assuming an expected proportion of welfare issues of 14%, with 5% absolute precision and 99.5% confidence interval (CI). The expected proportion of welfare issues was obtained by averaging the proportions of severe injuries and animals with poor welfare found by Marlin et al. [1,9,11] (i.e., 23%, 18.6%, and 0.1%, respectively). The minimum sample size required was 365 horses.

### 2.2. Experimental Protocol

Data collection was performed between May 2021 and April 2022, from 395 draft horses transported from different countries to the same Italian slaughterhouse (in Southern Italy) in 12 vehicles on 20 different dates (some vehicles were used multiple times, namely, vehicles ID 2, 4, and 5). All journeys were approved by the Competent Authorities at departure, double-checking that journey plans were realistic, the horses being transported were fit for transport, and the vehicles being used to transport the horses complied with current regulation. However, the fitness for transport of those horses was further assessed shortly before departure by one of the owners of the Southern Italian slaughterhouse, who has many years’ experience in horse breeding, transport, and slaughter. On arrival at the slaughterhouse, all measurements and assessments were carried out by the same veterinary surgeon, a freelance practitioner (FP) with extensive experience in assessing pre-slaughter health and welfare. The observations were performed three times, namely, during the unloading at the slaughterhouse, and within 30 min and 24 h after arrival, while the horses were kept in the lairage facilities. The welfare measures, including both animal-based measures (ABMs) and environmental-based measures (EBMs), were selected by a team of experts in the field of animal welfare and animal production at the University of Bologna using a literature review of horses’ welfare during and after road transport. A modified Messori et al. [9] protocol was developed. This protocol includes ABMs and EBMs to be recorded at driver-, vehicle-, and animal-level during unloading and lairage, plus the collection of transport documents (i.e., TRACES, Journey Log, International Consignment Notes—CMR, passports of the horses).

Table 1 shows the checklist used by the veterinarian during unloading procedures. The data collection started when the ramp was opened and ended when the last horse was unloaded (see Table S1 for details). The unloading was also recorded by another operator using a video camera (HDR-CX115E, Sony, Chengdu, China) for further analysis. Operators were not included in the recordings in order to protect their privacy.

Within 30 min of the unloading, the veterinarian inspected each horse kept in the lairage facilities following the checklist reported in Table 2 (see Table S2 for details and explanations of the measures).

Then, the veterinarian inspected the vehicle and asked questions about the driver’s experience and age, filling in the checklist reported in Table 3 (see Table S3 for details).

After the evaluation of the vehicle and the animals at unloading, copies of the Trade Control and Expert System (TRACES), Journey Log, Model 4, International Consignment Notes (CMR), and individual horse passports were collected for each journey and the relative load of horses examined. TRACES is mandatory for live animals transported across the borders of the Member States of the European Union and it is filled before the journey with the expected travel plan and health certificates to seek approval by the competent authorities. Journey Logs provide relevant information concerning the journey, such as the place and duration of stops, and whether the animals were fed during transport, and must be completed and submitted to the Local Authority for approval when animals are transported for more than 8 h by the driver. “Model 4” is a National (Italian) document, mandatory for the transport of live animals, comprising a declaration of origin that accompanies the animal during transport and contains information regarding pharmacological treatment or treatment with banned substances (hormones), details about the departure, destination, and transporter company names, and the health certificate of the veterinarian who examined the animal before movement (Italian Health Ministry). The International Consignment Note (Convention relative au contrat de transport international de marchandises par route; CMR), also known as International Road Carriage Waybill, is the standard contract of carriage of goods document and it is used when transporting goods and livestock animals internationally by road. CMR contains information about the scope and responsibility for the operation performed, identifies the parties involved and the goods being transported, and reports the weight of the goods (i.e., total weight of the load of animals) that are transported.

The passports of the horses were copied to obtain information about the animal’s country of origin and year of birth and to double-check the number of microchip for their identification. Microchips and passports have been mandatory in EU countries since 1 July 2009, when Regulation EC 504/2008 came into effect, and this is now enforced by Executive Regulation (UE) 2021/963. This regulation requires that all equines born in, or imported into the EU, must be identified using a passport and microchip. This regulation covers horses, ponies, donkeys, and other Equidae.

The day after arrival, the veterinarian returned to the lairage facility of the slaughterhouse. The environmental conditions of the lairage pens where the horses were housed were measured; for each pen, temperature, humidity, dimension, cleanliness of bedding, and the number of animals present in the pen/stabling box were recorded. The number of dead and exhausted horses at the lairage was also assessed, with any casualties recorded. Then, in order to evaluate the health and welfare of the animals, the veterinarian visually inspected each horse following the checklist reported in Table 4 (see Table S2 for details and explanations of the measures). After the inspection, all the horses were also tested using the Broken/Unbroken test (BUT) developed by Menchetti et al. [18]. Briefly, the test was composed of two behavioral tests, approaching and handling tests. During the approaching test, a handler gently approaches the horse and tries to apply the halter, and if haltering is successful, then during the handling test, the handler moves the horses three steps forward and three steps backwards (See Table S4 for details). While the handler was carrying out each test, the veterinarian scored the animal’s response. Horses that scored <2 were considered as unbroken.

Due to technical problems, it was not possible to gather information about the humidity upon arrival, while heart rates were never recorded as it was impossible to touch those horses. Deck and ramp height compliance was recorded but not measured.

### 2.3. Data Handling and Statistical Analyses

#### 2.3.1. Information Retrieved from the Gathered Documentation and Data Management

For each journey, the printed checklists (driver, vehicle, and individual animal) filled during unloading, 30 min, and 24 h after unloading at the lairage were double-checked against the copies of TRACES, Journey Logs, CMR, Model 4, and passports before being archived. At this step, additional information concerning the journey was also gathered from the documentation. TRACES and, when present, Model 4 were used to gather further information concerning the departure country, the estimated journey duration (reported on TRACES or Model 4), and, when reported, the total weight of the transported animals. Further information concerning the number and length of the stops during the journeys, the actual time of departures, and whether the animals were fed during transports, was obtained from Journey Logs. For each journey, the hours in transit were then calculated using departure and arrival hours to estimate the real duration of the journey, and then by subtracting the duration of the stops. The stops during the journey were categorized based on their length: stops were considered long when longer than 12 h and short when shorter. When present, the CMR consignment note was used to obtain the total weight of the transported animals. In case CMR was missing, the total weight of the load was obtained from TRACES.

Concerning the vehicle measurements taken at the slaughterhouse after unloading, the length and width of the internal part of the vehicle were used to estimate the total dimension of the internal part of the vehicle and the available space per animal. In detail, the thickness of the partitions used to divide the groups of horses inside the vehicle (each of which had a thickness of 3 cm) was subtracted from the length of the vehicle; the length was then multiplied by the width, and the obtained total space was divided over the number of animals transported to obtain the available space allowance (m^2^/animal). The space allowance variable was then dichotomized using as a threshold the minimum space allowance of 1.75 m^2^/horse suggested by Regulation EC 1/2005 [14] for adult horses (two classes for space allowance: “<1.75 m^2^/horse” or “≥1.75 m^2^/horse”). The obtained total space was also used to estimate the stocking density, expressed as a ratio between the total weight of the load and the actual total space available (kg/m^2^).

Data obtained from each journey were then transposed into an Excel file sheet. Each row was a journey, and columns comprised the data about the driver; the temperature at arrival; the characteristics of the vehicle and the ramp; the information retrieved for the journey from the documentation; the management of the unloading phase; and the presence and number of horses with severe health issues at unloading or that fell, slipped, or galloped at unloading. For each journey, the absolute numbers of animals that showed severe health issues or the aforementioned behavioral events at unloading were transformed to percentages of horses per vehicle showing a specific health issue or behavior.

For each horse, the two checklists with the individual information gathered at unloading and the following day in the lairage pens were double-checked and completed with information coming from the animals’ passports. In particular, passports were used to obtain the age, country of birth, and sex. The data for each individual were then transposed in an Excel file sheet where each row was a horse and each column contained information about its health and welfare status at unloading and the following day; the environmental conditions in the lairage pens; its scores for BUT and whether it was scored broken or unbroken. In the same Excel data sheet was also copied the information about the journey and vehicle each horse was transported on (all data reported for each journey and previously described were copied for the animals transported on the same vehicle).

#### 2.3.2. Statistical Analysis

The list of the variables included in the statistical analysis and their description is reported in Table 5. As diarrhea and abnormal feces had a low prevalence among the horses, they were combined into a unique variable.

To describe the dataset, descriptive statistics were performed using the Statulator^®^ online free software [20]. Data were reported as minimum (Min), maximum (Max), and mean values ± standard deviations (S.D.) for the continuous variables and reported as counts and percentages for the dichotomous and categorical variables.

To compare the health parameters observed in the transported horses within 30 min and 24 h after unloading, a two-tailed Student’s *t*-test was used for respiratory rate and two-tailed McNemar’s χ^2^ test statistics was used for categorical variables [21].

In order to identify associations between welfare issues and journey conditions, regression analysis was performed. Behavioral parameters observed at unloading were not used as outcome variables in the regression models because horses could not be individually identified accurately during unloading, only at the lairage. The dummy dependent variables of the regression models were presence/absence of diarrhea and abnormal feces and presence/absence of nasal discharge. The results of the univariate logistic regression are reported as odds ratio (OR), 95% CI, and *p*-values. The *p*-values were calculated using the Wald test, and for each outcome, the variables that showed a *p* < 0.250 were considered for inclusion in the stepwise multiple regression. A stepwise backward elimination procedure was indeed conducted for each dependent variable (presence/absence of clinical signs) to test the combined effect of the predictive variables. Collinearity between categorical variables was tested using Kendall’s tau statistic with the *cor.test* function in R environment or *glm* function for dichotomous variables [22]. The driver’s age and experience were collinear, and only the variable which resulted as more significant in the univariate single-variable model was kept in the following stepwise backward selection process to identify the final multivariable regression model. The category “partial” of the variable vehicle bedding was collinear with winter as the season, and for this reason vehicle bedding was not included in the multivariable regression models when season was significant. Similarly, ramp covering and the presence of ramp side gates were also collinear with winter as season and were not included in the multivariable regression models when season was significant. Collinearity was also found between the presence of clinical signs (health parameters) at unloading and 24 h after unloading. For example, the variable “presence of abnormal feces” at unloading was collinear with the presence of abnormal feces 24 h after unloading, as all the horses that showed abnormal feces at unloading had also this clinical sign 24 h after unloading. Similarly, nasal discharge at 24 h after unloading was collinear with nasal discharge at unloading, with all the horses that showed nasal discharge at unloading also having this health issue 24 h after unloading. Since only the clinical signs of abnormal feces and nasal discharge at 24 h after unloading had a percentage of affected animals around 5% or higher, they were used as dependent variables in univariable logistic regression models to identify possible risk factors associated with the occurrence of these clinical signs. The predictive variables were removed until all variables in the final model had a *p* < 0.100 or a lower Akaike information criterion (AIC) value than the other possible models. The final multivariable models resulting from the stepwise backward selection are presented as OR, 95% CI, and *p*-value. The scripts used to perform the univariate logistic and stepwise multiple regressions were a combination of functions in the packages *nlme*, *lsmeans*, *lme4*, and *car* in R environment [22].

## 3. Results

### 3.1. Description of the Routes

**Figure 1:**
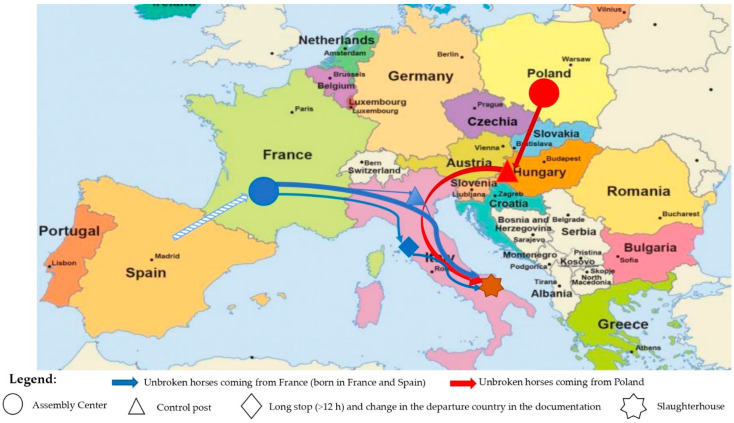
Journey routes of 395 unbroken horses travelling towards a slaughterhouse in Southern Italy.

France was the most frequent country of departure (13/20 journeys, 65%; 255/395 horses, 65%), followed by Poland (4/20 journeys, 20%; 79/395 horses, 20%) and Italy (3/20 vehicles, 15%; 61/395 horses, 15%). However, one out of the three journeys that departed from Italy had, together with Model 4, also the TRACES and the CMR consignment notes. Those documents attested that the horses actually originated from an Assembly Center located in France, and after a first transport from France to Central Italy, were unloaded and stabled there for about 16 h before being transported to the slaughterhouse in Southern Italy. The TRACES and Journey Logs were not available for the other two journeys that officially departed from Central Italy. The transport company was, however, the same for the three journeys, and the passports of the horses certified that all animals were born in France or Spain. It was therefore supposed that all these journeys originated from the same Assembly Center in France and had a similar journey duration. Based on the collected information, the actual departure countries of the 20 vehicles were France (16/20 vehicles, 80%; 316/395, 80%) and Poland (4/20 vehicles, 20%; 79/395 horses, 20%). Figure 1 summarizes the routes of the 20 journeys that transported the 395 horses included in the study.

The complete list of the journeys, routes, stops, and duration are reported in Table 6.

#### 3.1.1. Routes from France to Southern Italy

The journeys starting from France were 16/20, for a total of 316/395 horses (80%), and all originated from the same Assembly Center (Aveyron department, Occitania region, France).

Eleven journeys (11/16, 68.75%; 217/316 horses, 68.67%) lasted on average 24 ± 3 h (min = 19.40 h; max = 28 h). During the journey, two short stops of 1 h were performed. They stopped in Northern Italy (1/11, 9.1%), in Northern and Central Italy (4/11, 36.4%), in Northern and Southern Italy (4/11, 36.4%), in France and Northern Italy (1/11, 9.1%), or in France and Central Italy (1/11, 9.1%). The horses transported on this route had Spain (183/316, 57.91%) or France (133/316, 42.09%) as countries of origin.

The 3/16 (18.75%) journeys that had Central Italy as departure on Model 4 (61/316 horses, 19.30%), actually departed from France and traveled for about 14 h before the stop at a farm in Central Italy. Here, the horses were unloaded, watered, and fed. After 16–24 h of stop, the horses traveled for about 9 h to the slaughterhouse. Those horses were also born in Spain (17 out of the 61; 27.87%) or France (44/61; 72.31%).

One journey (1/16 journeys, 6.25%; 19/316 horses, 6.01%) lasted 12 h, from France to a control post (CP) in Northern Italy. After the compulsory stop of 24 h at the CP, the horses continued traveling for 11 h, with a short stop of 1 h in a rest area on the highway in Southern Italy. The transported horses were all born in France.

One journey (1/16 journeys, 6.25%; 19/316 horses, 6.01%) from France lasted 30.5 h. No information about the stops was available as Journey Log was not complete. The horses transported on this route had Spain as the country of origin.

The total distance from the assembly center to the slaughterhouse was about 1600 km. Of all the horses transported from France, a total of 219 were born in Spain (219/316, 69.30%). On the basis of the documentation, however, it was not possible to establish when the horses were moved from Spain to France.

#### 3.1.2. Routes from Poland to Southern Italy

Four journeys departed from an Assembly Center in Zaborów Drugi (Tomaszów Mazowiecki, Poland), traveled for 12–13.5 h, and then unloaded the horses at a CP in Hungary where the horses rested for a compulsory long stop (24–25 h). On the way to the CP, they stopped for a short stop (1 h) in Slovakia (1/4 journeys, 25%; 20/79 horses, 25.32%) or Czech Republic (1/4 journeys, 25%; 20/79 horses, 25.32%). For the remaining two, the location of the first short stop was unknown. After the long stop at the CP, the horses traveled over 16.5–19 h to reach the slaughterhouse, but in three cases (3/4 journeys, 75%; 59/79 horses, 74.68%), they stopped for 1 h in Northern Italy. For the remaining one, the location of the second short stop was unknown. The total distance from the assembly center to the slaughterhouse was about 2000 km. All horses transported on these routes had Poland as their country of origin.

#### 3.1.3. Documentation and Journey Duration

Table 7 reports the descriptive statistics for the journey duration declared on TRACES, the actual journey duration calculated from Journey Logs, and the hours in transit. The minimum journey duration declared from documentation was 8 h and was reported on the Model 4 for the three journeys that had Central Italy as the departure site. As these three vehicles came instead from France, their effective journey duration was about 40–48 h in total, and 25 h in transit. For four vehicles, it was not possible to calculate the actual journey duration and hours in transit, as a Journey Log was lacking. In one case it was not possible to assess whether the vehicle had a long stop, as a Journey Log was missing and the information was also omitted from TRACES. In all cases, the journey duration reported on TRACES was shorter than the actual duration of the journey, with an average travel time of 18 ± 8 h declared on TRACES and an actual duration of 34 ± 14 h calculated from Journey Logs. Concerning journey breaks, eight vehicles had a long stop (8/19; 42.10%). Among them, five stopped at a CP for 24 h (5/8; 62.50%), and three (3/8; 37.50%) stopped at the farm in Central Italy for about 16–24 h. Concerning short stops, most of the time there were two short stops while traveling (14/16; 87.5%), while one load stopped once (1/16; 6.25%), and thrice (1/16; 6.25%). All short stops lasted 1 h.

Concerning other documentation, passports were available for all the transported horses, and microchip numbers always matched the information reported on the passports and TRACES. On the other hand, CMR was available only for 5 out of the 20 journeys, and load weight was retrieved in most of the cases from TRACES, as animals were not weighed at unloading. Based on the passports, the transported horses were on average 2 years old ± 8 months, with a median of 2 years. The youngest horse transported was a foal of 6 months, and the oldest horse was 8 years old.

### 3.2. Description of Vehicles and Journey Conditions

**Figure 2:**
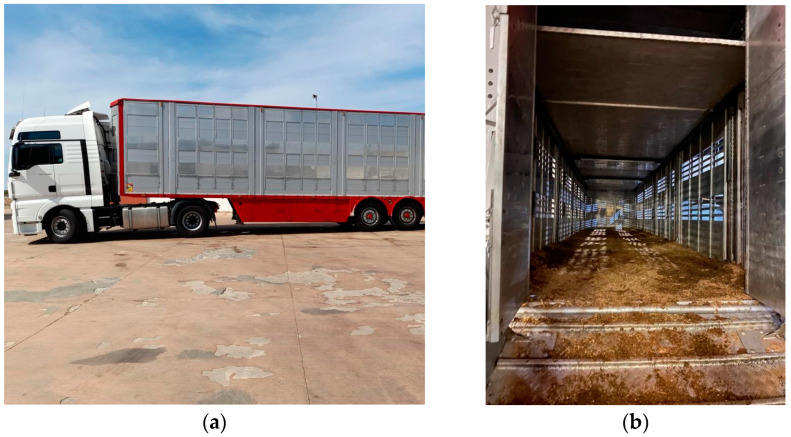
One of the vehicles arriving at the slaughterhouse in Southern Italy. (**a**) The truck and the straight-frame semi-trailer where horses were transported; (**b**) the interior of the vehicle observed after the horses were unloaded.

The 20 journeys took place in 12 different vehicles. All of the 12 vehicles were type-2 authorized vehicles, according to Council Regulation (EC) 1/2005 [14]. The vehicles were authorized for transport over long journeys, and thus were equipped with a light-colored roof, a ventilation system, removable partitions, a water tank, and the floor was covered with bedding material. All vehicles were also equipped with an internal lighting system for the care and inspection of animals during transport, and for loading/unloading procedures. The vehicles had also a temperature monitoring system, but due to the language barrier, it was difficult to find out from the driver if it really worked. In one case it was confirmed by the veterinarian that the temperature monitoring system was not working at all. Only one vehicle had cameras to monitor the animals during transport (1/12, 8.03%). All vehicles had a truck and a straight-frame semi-trailer (Figure 2).

Table 8 shows the descriptive statistics of the driver’s information and vehicle characteristics. All drivers were experienced, being involved in animal transport for at least 12 years, and had a competence certificate issued by competent authorities. Vehicles had an average dimension of 33.63 ± 0.56 m^2^ and transported on average 20 horses. The space allowance in most of the journeys (15/20; 75%) was below 1.75 m^2^/animal. Stocking density varied from a minimum of 266.30 kg/m^2^ to a maximum of 382.90 kg/m^2^.

Table 9 shows the frequency of vehicles displaying the different characteristics and the relative number of horses transported in those journey conditions. Horses were always transported on the same deck, and vehicles were all equipped with an adequate deck height. Partitions to stall horses were always present. All the horses were loose (i.e., free to move) and traveled in groups of three or four animals. On six journeys (6/20; 30%), stallions were transported in stalls adjacent to females or other stallions. The bedding quantity in the vehicle was sufficient to cover the vehicle flooring in the majority of the journeys (16/20; 80%). Straw was the most common bedding material. In most of the cases, the water tank was partially empty at arrival (14/20; 70%), and in two cases (2/20; 10%) it was empty. Drinkers were nipples (13/20; 65%) or bowls (7/20; 35%). Potentially harmful openings or sharp edges inside the vehicle were never noticed. Ramp flooring was most frequently made of non-slip knurled metal with foot battens (11/20; 55%), or rubber mats (6/20; 30%). Ramp lateral protections were present in the majority of vehicles (15/20; 75%).

### 3.3. Description of Unloading Conditions and Behavioural Parameters at Unloading

The unloading phase lasted on average 22 ± 5 min (minimum 15 min, maximum 37 min, median = 20 min) and the arrival temperature was on average 12.05 ± 7.04 °C (minimum −1 °C, maximum 27 °C, median = 10.5 °C). Horses self-unloaded and were directed towards lairage pens using voice and gestures (Video S1). No human–horse interactions were therefore registered, and no animals were slapped or moved inappropriately. No horses showed reluctance to move or fell during unloading. Three horses (3/395; 0.76%) galloped and jumped while being unloaded from the same vehicle, while 34 horses (34/395; 8.61%) that traveled on 12 journeys (average frequency for the 12 vehicles = 14.34 ± 3%) lost balance/slipped during unloading. No DOA, exhausted, or severely lame horses were recorded, and all horses were able to stand and walk unaided. Sweating and coughing were also never observed at unloading.

### 3.4. Descriptive Results for the Health Parameters Measured within 30 min from Unloading

The transported horses were all draft horses, mainly males (279/395; 70.63%), with an average BCS of 4 ± 0.5. The minimum BCS noticed was 3 and the maximum was 5. A BCS of 5 was observed in 60 horses (60/395; 15.19%). All horses were alert and responsive, or alert and calm. None of them wore a halter. On average, within 30 min from unloading, the horses had a respiratory rate of 20.5 ± 5.6 bpm, ranging from a minimum of 10 bpm to a maximum of 44 bpm. Horses were short-coated and did not show sweating signs. Tails were not injured nor were ruffled. Table 10 shows the prevalence of health parameters observed within 30 min after unloading. Diarrhea and abnormal feces were the clinical sign most frequently observed (26 horses out of the 395), with a prevalence of 6.58% on the total population. Ten journeys had at least one horse with diarrhea or abnormal feces, with a prevalence per journey ranging from 5% (1/20 horses transported) to 22.22% (4/18 horses). Six horses that traveled on six different journeys had injuries, mainly superficial cuts on the shoulders, legs, and trunk. Among the 17 horses displaying nasal discharge, only one had bilateral discharges, and they traveled on seven different journeys, with a prevalence per journey ranging from 5% (1/20 horses transported) to 19.05% (4/21 horses). Among the animals showing other types of discharges, lacrimal and genital discharges were found in five (5/395; 1.27%) and four horses (4/395; 1.01%), respectively. On the whole, 12 journeys had at least one horse with discharges, with the maximum number of horses having discharges noticed on a journey that departed from France during winter (5/21; 23.81%). Discharges were watery in all the observed cases. Among the seven horses displaying lameness, one animal showed a lameness score of 2 (1/395; 0.25%), while the others were scored as 1. The lame horses were from four different journeys, with one or two lame horses per journey. No horse displayed cough or signs of colic.

### 3.5. Descriptive Results for the Variables Measured 24 h after Unloading

During lairage, all the transported horses were housed in shaded stabling boxes, where they were free to move (untied) on clean bedding. The average temperature in the stabling boxes/paddocks was 12.76 ± 7.48 °C, ranging from a minimum of 2 °C to a maximum of 30 °C (median = 11 °C). The average relative humidity was 39.87 ± 17.02%, ranging from 10% to 70% (median = 43%). The slaughterhouse was provided with stabling boxes measuring 4 × 5 m, or with larger shaded paddocks of 10 × 9 m. In 4 × 5 stabling boxes were housed one (29/395; 7.34%), two (230/395; 58.22%), or three (12/395; 3.04%) horses at a time. The larger shaded paddocks housed between four and 11 horses; most frequently six or seven at a time (65/395; 16.56%). All horses were alert and responsive or alert and calm. The respiratory rate was 19.9 ± 5.1 bpm, ranging from a minimum of 10 bpm to a maximum of 44 bpm, and did not differ from the respiratory rate observed within 30 min after unloading (Student’s *t*-test *p*-value > 0.05). Table 11 shows the prevalence of health parameters observed 24 h after unloading. The prevalence of the health parameters remained stable between unloading and 24 h after unloading (McNemar’s χ^2^ test was not significant), and the horses showing the health issues at unloading displayed the same clinical signs also 24 h after unloading (correlation coefficient r > 0.90; *p*-value < 0.001). Similar to what was observed at unloading, only one animal had bilateral nasal discharge, and among the animals showing other types of discharges, lacrimal and genital discharges were found in five (5/395; 1.27%) and four horses (4/395; 1.01%), respectively. Discharges were watery in all the observed cases. Lameness was never severe, with five (5/395; 1.27%) out of the seven horses being scored as 2, and the remaining two (2/395; 0.51%) being slightly lame (score 1).

All but five horses were declared to be unbroken. Their level of tameness was confirmed by the results of the BUT test, which scored 390/395 horses as unbroken and confirmed the five horses being broken (BUT score > 2). A recording of a BUT performed on an unbroken horse in the lairage pens is shown in Video S2.

### 3.6. Risk Factors Associated with the Presence of Diarrhea or Abnormal Feces 24 h after Unloading

Table 12 shows the OR, 95% CI, and *p*-values of the variables significantly associated with the presence of diarrhea and abnormal feces in the univariable logistic regressions. Only the significant associations and/or the results of the variables that entered in the final multiple regression model were retained in the table. The complete list of *p*-values obtained from univariable logistic regressions for all the independent variables is reported in Table S5. When considered separately, lower arrival temperatures, winter as transport season, no provision of food during transport, lower stocking densities, and younger drivers were significantly associated with increased odds of having horses with diarrhea or abnormal feces 24 h after unloading. Taken individually, the odds of finding horses with abnormal feces 24 h after unloading were increased by six times when the horses were transported in winter and by more than two times when they were not fed during transport. A trend towards significance was also noticed for the variable of space allowance classes.

Table 13 shows the OR, 95% CI, and *p*-values of the variables retained in the final multiple regression model for the presence of diarrhea and abnormal feces (model *p*-value = 0.003). The season was strongly associated with the presence of diarrhea and abnormal feces (*p* < 0.001), with horses transported in winter being almost six times more likely to display this health parameter 24 h after unloading. The variable of space allowance classes was retained in the model as the AIC obtained including this variable in the multiple regression model was smaller (AIC = 178.9) than the AIC of the model without space allowance classes (AIC = 179.15). Larger space allowances seem, therefore, to increase the odds of finding horses with diarrhea and abnormal feces when journeys take place during winter.

### 3.7. Risk Factors Associated with the Presence of Nasal Discharge 24 h after Unloading

Table 14 shows the OR, 95% CI, and *p*-values of the variables significantly associated with the presence of nasal discharge in the univariable logistic regression models. Only the significant associations and/or the results of the variables that entered the final multiple regression model were retained in the table. The complete list of *p*-values obtained from univariable logistic regressions for all the independent variables is reported in Table S6. When considered separately, winter as transport season, no provision of food during transport, and less experienced drivers were significantly associated with increased odds of having horses with nasal discharge. A trend toward significance was also noticed for the presence of long stops during the journey.

Table 15 reports the estimates, OR, 95% CI, and *p*-values of the variables retained in the final multiple regression model for the presence of nasal discharge 24 h after unloading (model *p*-value = 0.027). The predictive variables of “fed during transport” and the arrival temperatures were strongly associated with the presence of nasal discharge, with horses that were transported when the arrival temperature was lower and that were not fed during transport being almost six times more likely to display this health problem. The variable of space allowance classes was retained in the model, as the estimated AIC including this variable in the multiple regression model was smaller (AIC = 120.64) than the AIC of the model without space allowance classes (AIC = 121.07). A smaller space allowance seems, therefore, to increase the odds of finding horses with nasal discharge when they are not fed during transport and at arrival at the slaughterhouse temperatures were low.

## 4. Discussion

This study described for the first time the routes and the journey conditions of unbroken/unhandled horses traveling across the European Member States towards a slaughterhouse in Southern Italy and documented their welfare status on arrival using an objective, evidenced-based protocol. Associations between journey conditions and welfare issues were identified, supporting the hypothesis that journey conditions are crucial for the welfare of unbroken horses. Surprisingly, the prevalence of the welfare issues of those horses were very low even when those horses were transported for more than 8 h. This was probably due to the fact that, apart from journey durations, the horses traveled in compliance with the requirements requested by the EC 1/2005 for this horse category [14]. Our findings are useful to demonstrate that the welfare of unbroken horses can be protected during the journey when they can travel in small groups of familiar horses, loose and with enough space to balance, in adequate vehicles (Type 2 authorization), and undertake journey breaks, either short food and water stops inside the vehicles or long rest stops at control posts. This study provides evidence that may help policy-makers in implementing the current regulation on the protection of the welfare of horses during transport.

Information on the routes and journey conditions of unbroken horses traveling across European member states is scant [8]. In our study, the destination was a slaughterhouse in the South of Italy, where horse meat is eaten as a traditional meal [23]. According to Russo et al. [24], the consumption of horse meat in Italy in 2010 was 0.75 kg per capita and fell to 0.52 kg per capita in 2015. However, in line with the literature [19,25], horse meat comes from local Italian horse breeds in fewer than half of the cases, and mainly comes from horses born and reared in Spain, France, and Poland, as in our dataset. Data from 2017 showed that Poland was indeed among the top five producers of horsemeat in the EU [2]. In Spain, horses are kept in extensive systems, grazing the natural resources provided by mountain areas. The horses kept in this way are usually unbroken and not used to human interactions and handling and are shipped to other countries for slaughter [26]. A trade of Spanish unbroken horses was identified in the literature, and they were named the “invisible horses” [6,7] because they are often untracked animals, which are transported for more than 8 h as if they were broken (EC 1/2005) [14]. In our dataset, many horses had Spanish passports, and consequently could be part of the aforementioned trade. However, those horses arrived in France before the journey we monitored in our study, and we had no information related to the first leg of their journeys. The average duration declared on the TRACES of the monitored journeys was lower than the real duration calculated from the Journey Log. This is a common problem and a limitation of the current TRACES monitoring system. Another limitation of TRACES emerges from this study. Even if the EC 1/2005 set a different journey duration depending on the level of tameness of the horse, it is worth noting that TRACES for horses does not have any criteria related to the level of tameness. Consequently, the competent authorities approving the journeys and then receiving the TRACES at destination are unable to double-check whether the journey duration is appropriate for the level of tameness. In our study, all horses traveled over long distances and their TRACES were indeed approved, even if the horses were unbroken. However, apart from journey duration, the horses assessed in our study traveled following the requirements for unbroken horses, namely, traveling loose and in small groups, in most cases fed and watered every 8 h during short or long stops, in well-equipped vehicles, driven by experienced drivers, stopping frequently, and with a stocking density of about 330 kg/m^2^. Overall, they arrived in good health and welfare conditions, demonstrating that journey conditions may be more important than journey duration in itself.

Unloading lasted approximately 20 min, and the horses self-unloaded. They showed minimal behavioral problems, e.g., refusing to unload and rushing off [27], and balance-related issues, e.g., slipping and falling. Self-loading and self-unloading training is highly beneficial for meat horses since it reduces the fear and the stress associated with these procedures and it also reduces the duration of those procedures, the occurrence of human–horse interactions [28], and the consequent risk of horse-related human injuries [29]. This practice should be strongly recommended considering that poor handling is listed among the most important hazards of the highly relevant welfare consequences caused by horse transportation [8], and self-unloading completely prevents any handling. Interestingly, no signs of sweat were observed during unloading. Sweat is considered a useful welfare ABM at unloading because horses sweat not only due to heat but also due to agitation for the prolonged waiting time or inappropriate handling [8]. It is worth noting that the external environmental temperature at arrival was only once above the upper critical temperature of the thermo-neutral zone [30], and that when the environmental temperature was high, the horses travelled overnight. Considering the lack of DOA, exhausted animals, severely lame and injured horses, and also the lack of sweating, coughing, and other negative behavioral ABMs, it can be concluded that the welfare status of those horses was of an acceptable standard even after a long journey, which was managed ensuring good journey conditions.

The welfare assessment within 30 min showed that the most common health problems were diarrhea and abnormal feces, which were also present in half of the journeys. This is in agreement with the literature, where long transports were found to significantly increase the average prevalence of pathogens in feces [31,32]. The second most common health problems were nasal and other discharges, which instead were present only in a third of the journeys. This is in line with the total prevalence of 33% of ocular and nasal discharge observed in 2648 horses slaughtered in Northern Mexico [11]. Lameness was lower than expected, considering that those horses traveled loose, as it is often wrongly believed that the lack of restraining can increase the risk of falling and slipping inside the vehicle. However, this prevalence is in line with those reported by Messori et al. [9] for handled horses and Roy et al. [33] for unhandled horses. The welfare assessment carried out 24 h after arrival showed a similar prevalence, demonstrating that the animals were not incubating any disease. Transport-related diseases may indeed manifest some hours/days after arrival and lead to death afterward. In our case, there were no dead or exhausted animals at the lairage, confirming the findings of the welfare assessment during and soon after unloading. Our study further validated the use of the BUT test to differentiate the level of tameness of a horse. Menchetti et al. [18] applied the test on 100 horses on-farm, where they were usually kept. During this study, a further 395 horses were also tested in a new environment, and the BUT scores and classifications coincided with the declaration of the owners. The BUT test should become a practice before loading the animals, to adapt the journey conditions to their level of tameness.

The prevalence of injuries was surprisingly low (1.7%). It was indeed much lower than the prevalence reported by Marlin [1], where 17% of horses at an assembly area in Romania and 24% of horses at slaughter plants in Italy had at least one recent injury, showing that the lack of fitness for transport was a welfare concern for horses destined for slaughter. However, our injury prevalence is in line with what was registered by Roy et al. [33] in Iceland, when the authors assessed the welfare of adult horses and foals transported in similar conditions (small groups, loose). In the latter study, all the horses were checked by the authors before the journeys and only the animals fit for transport were loaded. In our study, the fitness for transport was routinely and fully assessed before loading by one of the owners of the slaughterhouse, who had the good practice of not loading horses with injuries or any other clinical signs, in agreement with the recent European guidelines [34]. For safeguarding horse welfare during transport, a correct assessment of the fitness for transport is strongly recommended, because the pain associated with any preexisting injuries or disease will affect the horse’s emotional state and become increasingly worse during the journey [8].

Even if horses were fit for transport, gastroenteric disorders were the most common health problems at unloading and in lairage. Transport stress can lead to gastroenteric problems [35], which may vary from a simple reduction of the gastro-enteric motility [36] to stomach ulcerations [37]), and from diarrhea [38] to colic [39], including fatal enterocolitis (i.e., salmonellosis, and clostridiosis) [32,35,40]. Diarrhea was reported by 20% of the participants of a survey on transport-related issues conducted in Australia, and the odds of reporting this problem were lower when the participants were professionals (i.e., people who gain money from horses) moving horses commercially [41]. In this study, all horses were moved during commercial transportation by very experienced drivers, and this could be the reason for the lower prevalence of abnormal feces and other gastro-enteric problems, including colic observed. The driver’s age was also found to be negatively associated with the presence of abnormal feces, confirming the importance that horses must be moved by experienced staff with excellent driving skills [8]. The lack of feeding was also associated in the univariable models, confirming that long fasting is a risk factor for gastroenteric disorders related to transport and that horses should be fed in transit or at least during the short stops [8,42]. In the multivariable model, abnormal feces remained associated only with season and space allowance. Winter seasons increased, by almost six times, the likelihood of finding this problem. This could be due to the fact that in winter, temperatures are lower and more viruses and bacteria could have been shed, leading to diarrhea [43]. In winter, temperatures were often between −1 and 10 °C upon arrival in Southern Italy, so it could be supposed that the temperature was much lower in France and during the journey, considering that when horses do not eat ad libitum in transit they are unable to produce heat by eating and digestion. Consequently, it could be assumed that the temperature inside the vehicle during the journey was lower than the comfort thermal zone (approximately 5 °C) [30]. The odds of abnormal feces increased when the space allowance was higher, possibly because in that case fewer horses were inside the vehicle, so less heat was generated and cold wind could easily circulate around them, affecting their gastro-enteric motility. Horses should travel within their thermal comfort zone inside the vehicle to minimize thermal stress in transit and its possible consequences [8].

Similarly, nasal discharge was associated with cold temperatures at arrival, lack of feeding stops, and limited space allowance. Transport can lead to respiratory disorders, from inflammation of the upper respiratory tract [44] to an increase in tracheal mucus [45] and pleuro-pneumonia [46]. Time spent with the head and neck below the wither height was identified as a risk factor for increased tracheal mucus and pneumonia [45,47]. When horses cannot lower their head to the floor level the mucociliary movements decrease and bacteria and mucus tend to accumulate in the lower respiratory tract [48]. The prevalence of respiratory problems in our study was lower than those reported in the literature [40,49,50]. This could be related to the fact that these horses travel loosely, while higher prevalence was reported in studies where horses traveled restrained (i.e., cross-tied, or tied short) [51]. The lack of feeding was associated with higher nasal discharge; this could be because when horses are not fed in transit, they may spend more time with their heads in elevated position. Having the hay on the floor could be indeed a good motivation to spend time eating with the head down, allowing the clearance of the respiratory system [45,52]. The association with the reduced space allowance may have a similar explanation since lack of space could have limited the horses in their ability to balance and move their heads down [53,54]. Furthermore, a smaller space available per horse could have led to increased shedding of respiratory tract pathogens, as a greater number of animals found themselves at close distances from possible subjects capable of transmitting the pathogens. Increased contact between individual shedding and susceptible horses, such as increased number of nose-to-nose interactions and more animals exposed to fomites, are risk factors for pathogen spread [55]. Variation of temperature between departure and arrival was identified as a risk factor for bovine respiratory diseases after transport [56]. This has not been investigated in horses yet. However, cold temperature and transport stress could have facilitated the spread of the winter respiratory virus and led to increased odds of nasal discharges, as demonstrated in beef steers [56]. Our findings confirm that for minimizing the incidence of transport-related respiratory problems, horses should be transported in a manner allowing the head and neck to move freely and with adequate space allowance [8].

Our results need to be interpreted with caution since the study had several limitations. The main limitation was the fact that data were recorded only at one slaughterhouse. This was performed to standardize the method of assessments, but clearly the prevalence of the welfare problems cannot be generalized to all horses transported to slaughterhouses, since it may reflect the good practice and the long-term experience of the drivers and slaughterhouse staff who moved the horses used in this study. Some documents were missing or sometimes documentation was not complete, so some data were missing. This in particular affected stocking density, which can consequently be considered only indicative. Finally, no diagnoses of the health problems were confirmed using laboratory tests, since the main scope of this study was to use a protocol containing EBMs and ABMs which could be considered feasible and repeatable. Notwithstanding those limitations, this study is the first to report details concerning the journeys and the welfare status recorded with a standardized protocol during and after unloading of unbroken horses moved across Europe. The findings may be useful to suggest best practices, guidelines, and preventive measures to safeguard the welfare of unhandled horses during their journeys.

## 5. Conclusions

This study described for the first time the journeys and the journey conditions of unbroken/unhandled horses across European Member states. No mortality or other severe health problems were recorded during unloading and the lairage period. Only a few horses showed minor injuries, and the prevalence of abnormal feces and nasal discharge was about, or slightly above, 5%. For those health problems, the limited experience and young age of the drivers, space allowance, lack of feeding, and the environmental conditions were risk factors, confirming our hypothesis. Journey conditions, including a correct assessment of fitness for transport and traveling loose in small groups, seem to be crucial to safeguard the welfare of unbroken horses during traveling. Further studies on the ideal space allowance/stocking density and environmental conditions, such as the possibility to travel in a fully conditioned vehicle, are needed to suggest requirements for future regulation.

## Figures and Tables

**Table 1:** Checklist used **during unloading** to evaluate the welfare status of horses at arrival in a Southern Italy slaughterhouse (modified from Messori et al. [9]).

	Variable	Observations
Unloading Phase: one copy for each journey	Arrival temperature and humidity	_ _ _ _ _ _
	Date and time of arrival	_ _ _ _ _ _
	Duration of unloading	_ _ _ _ _ _ _ _ min
	Total animals transported	_ _ _ _ _ _ _ N horses
	Dead on arrival (DOA)	_ _ _ _ _ _ _ N horses
	Non-ambulatory *	_ _ _ _ _ _ _ N horses
	Reluctant to move *	_ _ _ _ _ _ _ N horses
	Severely lame *	_ _ _ _ _ _ _ N horses
	Slipping/losing balance	_ _ _ _ _ _ _ N horses
	Falling	_ _ _ _ _ _ _ N horses
	Galloping or jumping	_ _ _ _ _ _ _ N horses
	Body Condition Score	Score 0–1: _ _ _ N horses Score 5: _ _ _ N horses
	Sweating	_ _ _ _ _ _ _ N horses
	Severe injuries	_ _ _ _ _ _ _ N horses
	Exhaustion	_ _ _ _ _ _ _ N horses
	Coughing (more than one event)	□ Yes □ No
	The handler moves the animals in an arousing manner, inducing fear	□ Yes □ No
	The handler moves the animals in a noisy manner, inducing fear	□ Yes □ No
	The handler hits the animals without reason	□ Yes □ No
	The handler moves the animals properly **	□ Yes □ No
	The handler performs prohibited practices **	□ Yes □ No

* Mutually exclusive; ** mutually exclusive; “non-ambulatory” = unable to independently assume and maintain a standing position; “reluctant to move” = unwillingness to move forward or move from own position; “severely lame” = obvious attempt to take the weight off one or more limbs; “losing balance” = slipping without the body hitting the ground; “severe injuries” = damage to deeper tissues, beyond the skin.

**Table 2:** Checklist used **within 30 min after unloading** of the last animal to evaluate the welfare status of the individual horses at the lairage facilities in a Southern Italy slaughterhouse (modified from Messori et al. [9]).

Date________ Hour________ Venue________ Vehicle ID________________ Horse ID________ Hours of Travel________ Horse Origin_________________	
Sex	□ Stallion □ Gelding □ Female
Halter	□ Yes □ No
Age	_ _ _ _ _ _ _ _
Horse type	□ Draft □ Light
Body Condition Score	_ _ _ _ _ _ _ _
Estimated weight	_ _ _ _ _ _ _ _
Demeanor	□ Alert, responsive □ Alert, quiet □ Lethargic □ Depressed □ Exhausted □ Terrified
Heart rate	_ _ _ _ _ _ _ _
Respiratory rate	_ _ _ _ _ _ _ _
Coat	□ Long □ Short
Sweating	□ Yes □ No
Feces	□ Normal □ Not formed □ Diarrhea
Clinical signs of colic	□ Yes □ No
Tail	□ Normal □ Ruffled □ Visible skin
	□ Abrasions, with damaged skin
Nasal discharge	□ Bilateral □ Unilateral □ None
Type of nasal discharge	□ Watery □ Purulent □ Bloody mucus □ Smelly
Other discharges	□ Eyes □ Mouth □ Rectum □ Vulva or penis □ None
Cough	□ Yes □ No
Cuts or injuries	□ No □ Yes (How many_ _ _ _ _ _Which_ _ _ _ _ _)
Lameness score	□ 0 □ 1 □ 2 □ 3 □ 4 □ 5
Other clinical signs	□ Yes_ _ _ _ _ _ _ _ □ No
Notes	_ _ _ _ _ _ _ _

**Table 3:** The checklist used after unloading to gather information about the driver’s age and experience, vehicle and ramp characteristics, and documentation at arrival in a Southern Italy slaughterhouse (modified from Messori et al. [9]).

	Variable	Observations
Driver	Age	_ _ _ _ _ _ _ _
	Years of experience	_ _ _ _ _ _ _ _
	Certificate of competence ^∫^	□ Yes □ No
Vehicle and Ramp	Date________ Hour________ Venue________ External temperature________ Humidity________ Vehicle ID________ Vehicle origin________	
	Vehicle covering	□ Yes □ No
	Forced ventilation	□ Yes (number)_ _ _ _ □ No
	Cameras	□ Yes (number)_ _ _ _ □ No
	Horses in group:	□ Yes Group number_ _ _ _ □ No Rope length (cm) _ _ _ _ □ Appropriate □ Inadequate
	If tied:	
	Deck height	(cm) _ _ _ _ _ □ Appropriate □ Inadequate
	Ramp height	(cm) _ _ _ _ _ □ Appropriate □ Inadequate
	Direction of travel (for horses travelling in single bay)	□ 90° (head left, right), □ 45° (head left, right) □ Forward, □ Backwards
	Adjacent stalls *	□ Yes □ No
	Ramp flooring type	_ _ _ _ _ _ _ _
	Ramp flooring conditions	□ Appropriate □ Inadequate
	Ramp covering	□ Bedding completely covering □ Bedding partially covering □ None
	Ramp lateral protections	□ Yes, without openings or sharp edges
		□ Yes, with openings or sharp edges □ No
	Slots/Steps (>10 cm between vehicle/ramp/floor/doors)	□ Yes □ No
	Ramp slope	□ Compliant □ Not compliant
	Blockage zones (holes or physical obstacles) on the ramp	□ Yes □ No
	Lighting for animal orientation	□ Yes □ No
	Lighting for animal movement during unloading	□ Yes □ No
	Sharp edges	□ Yes □ No
	Number of partitions	_ _ _ _ _ _ _ _
	Dimensions of bays/compartments	Width_ _ _ _ _ _ _ Length_ _ _ _ _ _ _ _
	Dimensions of the vehicle (m^2^)	_ _ _ _ _ _ _ _
	Vehicle floor covering	□ Bedding completely covering □ Bedding partially covering □ None
	Drinkers	Total number_ _ _ _ Working number_ _ _
	Drinker type	_ _ _ _ _ _ _ _
	Water tank	□ Full □ Partially Empty □ Empty
	Temperature monitoring and control systems	□ Yes, working_ _ _ _ _ _ _ _ (°C) □ Yes, not working □ None
	Signs of diarrhea in the vehicle	□ Yes □ No
	Documents/information checklist	□ Passport, □ CMR, □ TRACES, □ Journey Log, □ Weight of the load

* Presence of horses in adjacent bays that cannot travel close together safely (mare/stallion or stallion/stallion). ^∫^ Certificate of competence issued by the competent authorities as requested in the EC 1/2005.

**Table 4:** Checklist used **24 h after unloading** to evaluate the welfare status of the individual horses that arrived in a Southern Italy slaughterhouse.

Date________ Hour________ Venue________ Vehicle ID________ Horse ID________ Hours of Travel________ Horse origin________	
Paddock/stabling box temperature	_ _ _ _ _ _ _ _
Paddock/Stabling humidity	_ _ _ _ _ _ _ _
Paddock/stabling exposure	□ Sun □ Shadow
Number of horses per paddock/box	_ _ _ _ _ _ _ _ horses
Bedding cleaning	□ Clean □ Partially clean □ Dirty
Freedom of movement	□ Yes □ No
Sex	□Stallion □ Gelding □ Female
Age	_ _ _ _ _ _ _ _
Horse type	□ Draft □ Light
Declared level of animal handling	□ Broken □ Unbroken
Demeanor	□ Alert, responsive □Alert, quiet □ Lethargic □ Depressed □ Exhausted □ Scared
Heart rate	_ _ _ _ _ _ _ _
Respiratory rate	_ _ _ _ _ _ _ _
Feces	□ Normal □ Not formed □ Diarrhea
Clinical signs of colic	□ Yes □ No
Nasal discharge	□ Bilateral □ Unilateral □ None
Type of nasal discharge	□ Watery □ Purulent □ Bloody mucus □ Smelly
Other discharges	□ Eyes □ Mouth □ Rectum□ Vulva or penis □ None
Cough	□ Yes □ No
Cuts or injuries (including tail)	□ No □ Yes (How many_ _ _ _Which_ _)
Lameness score	□ 0 □ 1 □ 2 □ 3 □ 4 □ 5
Other clinical signs	□ Yes_ _ _ _ _ _ _ _ □ No
Broken/unbroken test (BUT)	Approaching and haltering □ 0 □ 1 □ 2 Handling □ 0 □ 1 □ 2
Notes	_ _ _ _ _ _ _ _

**Table 5:** Definition and type of the variables obtained for the present study.

Variables	Meaning	Types of Variables
Arrival temperature (°C)	External environmental temperature measured through a weather station.	Continuous
Unloading duration (min)	From the lowering of the vehicle ramp until the last horse was unloaded.	Continuous
**Routes**		
Country of origin	Country quoted in the passport as the horse’s place of birth (i.e., France, Spain, Poland).	Categorical
Country of departure	Country quoted in the Journey Log/TRACES as the horse’s country of departure (i.e., France, Italy, Poland).	Categorical
Short stops (<12 h)	Number of short stops (<12 h) during the journey without horses unloading.	Categorical
Long stops (>12 h)	Presence/absence of long stops (>12 h) during the journey.	Dichotomous
**Journey information**		
Duration from TRACES (h)	Journey duration from departure to arrival (from TRACES).	Continuous
Duration from Journey log (h)	Journey duration from departure to arrival (from Journey Log).	Continuous
Hours in transit (h)	How many hours the horses spent on the vehicle in motion (stops excluded).	Continuous
**Driver’s information**		
Driver’s age (years)	Age of the driver of the vehicle.	Continuous
Driver’s experience (years)	Years of experience driving vehicles for the transport of horses to the slaughterhouse.	Continuous
**Vehicle characteristics**		
Total vehicle dimension (m^2^)	Internal dimensions of the vehicles.	Continuous
Drinkers (*n*)	Number of drinkers on the vehicles.	Continuous
Horses per truck (*n*)	Number of horses on the vehicles.	Continuous
Space allowance (m²/animal)	The m² available for each horse.	Continuous
Stocking density (kg/m²)	How many kg of live weight are distributed per m².	Continuous
Load weight from CMR (kg)	Total weight of horses on a vehicle (from CMR).	Continuous
Load weight from TRACES (kg)	Total weight of horses on a vehicle (from TRACES).	Continuous
**Vehicle characteristics**		
Ramp flooring	Cover material for the unloading ramp (i.e., non-slip knurled metal with foot battens; corrugated metal; rubber mat).	Categorical
Ramp lateral protections	Presence or absence of lateral protections on the ramp.	Dichotomous
Ramp covering	Degree of coverage of the ramp with bedding material (i.e., none, partial, complete).	Categorical
Vehicle bedding	Degree of coverage of the vehicle floor with bedding material (i.e., partial, complete).	Dichotomous
Vehicle bedding type	Bedding material for the vehicle floor (i.e., straw, shavings, straw and shavings, sand and shavings).	Categorical
Group size	Number of horses per group (i.e., 3 or 4).	Dichotomous
Adjacent stalls	Presence of horses that cannot travel close together safely (mare/stallion or stallion/stallion).	Dichotomous
Type of drinkers	Structure and characteristics of the drinkers (i.e., portable, bowls, nipples).	Categorical
Water tank	Filling level of the water tank (i.e., full, partially empty, empty).	Categorical
Temperature monitoring andcontrol systems	Presence or absence of vehicle interior temperature control system.	Dichotomous
Fed during transport	Presence or absence of the feeding of horses during the journey.	Dichotomous
Long stops (>12 h)	Presence or absence of a stop during the journey with the horses unloaded from the vehicle, for feeding and watering.	Dichotomous
Number of short stops (<12 h)	Number of short stops (<12 h) during transport.	Continuous
Season	Season in which horses travelled (i.e., spring, summer, autumn, winter).	Categorical
Space allowance classes	The m² available for each horse, net of partitions, divided into classes (i.e., <1.75 m^2^/animal, >1.75 m^2^/animal).	Dichotomous
**Health parameters**		
Dead on arrival	Presence or absence of dead horses on the vehicle.	Dichotomous
Non-ambulatory	Presence or absence of horses unable to independently assume and maintain a standing position.	Dichotomous
Injuries	Presence or absence of injuries more or less severe on the horse’s body.	Dichotomous
Nasal discharge	Presence or absence of loss of material from the nose.	Dichotomous
Other discharges	Presence or absence of loss of material from other orifices (i.e., eyes, penis, vagina etc.).	Dichotomous
Respiratory Rate	Number of respiratory acts in one minute.	Continuous
Diarrhea and abnormal feces	Presence or absence of feces with altered consistency.	Dichotomous
Lameness	Presence or absence of a slight or modest attempt to take weight off one or more limbs.	Dichotomous
**Behavioral parameters at unloading:**		
Reluctance to move	Prevalence of horses per journey showing unwillingness to move forward or move from own position during unloading.	Continuous
Slipping/Loss of balance	Prevalence of horses showing uncontrolled limb movement due to slippery ground during unloading.	Continuous
Falling	Prevalence of horses showing loss of balance with body touching the ground during unloading.	Continuous
Galloping and jumping	Prevalence of horses galloping and/or jumping from the ramp during unloading.	Continuous
Demeanor	State of the sensorium and reactivity to external stimuli (i.e., alert/responsive, alert/quiet, lethargic, depressed, exhausted, scared).	Categorical
**Lairage characteristics**		
Temperature	External environmental temperature measured through a weather station.	Continuous
Relative humidity	External environmental relative humidity measured through a weather station.	Continuous
Dimensions	Width and length, in m^2^ (i.e., 4 × 5, 10 × 9).	Dichotomous
Number of horses per facility	Number of horses in stabling boxes (i.e., 1, 2, 3) or paddocks (i.e., 4–11, more frequently 6–7).	Categorical
Health parameters	The health parameters observed within 30 min from unloading and 24 h after unloading were expressed both as presence/absence and as prevalence of horses showing the health issue per journey.	Dichotomous and continuous

**Table 6:** Description of each journey, reporting the departure and arrival country, the country of birth of the horses, and the information about the journey (number and duration of stops; duration of the journey). * = Travel hours assumed from other documentation, not from Journey Log.

Journey ID	Vehicle ID	Route	Origin of Horses	Long Stops	Short Stops	Journey Duration (h)	Hours in Transit (h)	Arrival Time	Arrival T (°C)	Notes
1	1	FRA—ITA	ESP/FRA	1 (16 h) ITA	0	39 *	23 *	8:00	19	The horses born in Spain may have been reared in Spain or in France.
2	2	POL—ITA	POL	1 (24 h) HUN	2 (1 h each), 1st: SVK, 2nd: missing	55	29	8:00	24	The location of the second short stop is unknown.
3	3	FRA—ITA	FRA	Missing	Missing	39 *	23 *	8:10	25	Based on the available documentation, this journey was similar to Journey 1.
4	3	FRA—ITA	ESP/FRA	Missing	Missing	39 *	23 *	8:00	27	Based on the available documentation, this journey was similar to Journey 1.
5	4	POL—ITA	POL	1 (25 h) HUN	2 (1 h each), 1st: missing, 2nd: ITA	58	31	2:30	−1	The location of the first short stop is unknown.
6	5	FRA—ITA	ESP	0	2 (1 h each), 1st: ITA, 2nd: ITA	28.30	26.30	23:30	6	None.
7	5	FRA—ITA	ESP	0	2 (1 h each), 1st: ITA, 2nd: ITA	28	26	16:00	8	None.
8	6	FRA—ITA	ESP/FRA	0	2 (1 h each), 1st: ITA, 2nd: ITA	21.30	19.30	8:00	7	None.
9	4	FRA—ITA	FRA	1 (24 h) ITA	1 (1 h) ITA	48 *	23 *	9:15	11	Inconsistency between departure time on Journey Log and Traces.
10	7	FRA—ITA	ESP	0	2 (1 h each), 1st: ITA, 2nd: ITA	21.40	19.40	10:00	6	None.
11	8	FRA—ITA	ESP	Missing	Missing	30.30 *	Missing	23:30	8	The Journey Log is not completed.
12	9	POL—ITA	POL	1 (24 h) HUN	2 (1 h each), 1st: CZE, 2nd: ITA	57.30	31.30	20:00	10	None.
13	8	FRA—ITA	ESP/FRA	0	2 (1 h each), 1st: ITA, 2nd: ITA	23	21	11:30	15	None.
14	10	FRA—ITA	ESP/FRA	0	2 (1 h each), 1st: ITA, 2nd: ITA	27	25	19:00	8	None.
15	5	FRA—ITA	ESP	0	2 (1 h each), 1st: ITA, 2nd: ITA	20.30	18.30	15:00	11	None.
16	9	FRA—ITA	ESP	0	2 (1 h each), 1st: ITA, 2nd: ITA	24	22	17:30	12	None.
17	11	POL—ITA	POL	1 (24 h) HUN	2 (1 h each), 1st: missing, 2nd: ITA	57	31	2:00	10	The location of the first short stop is unknown.
18	12	FRA—ITA	ESP/FRA	0	2 (1 h each), 1st: FRA, 2nd: ITA	25	23	20:00	8	None.
19	12	FRA—ITA	ESP/FRA	0	2 (1 h each), 1st: FRA, 2nd: ITA	19.40	17.40	15:00	13	None.
20	9	FRA—ITA	ESP/FRA	0	2 (1 h each), 1st: ITA, 2nd: ITA	24.10	22.10	17:30	14	None.

**Table 7:** Descriptive statistics for the journey durations and load weight observed from the documentation of the 20 journeys of unbroken horses towards a Southern Italy slaughterhouse.

Journey Duration	Mean ± S.D.	Min	Median	Max	Vehicles with Missing Information
Duration from TRACES (h)	18 ± 8	8	17	48	2
Duration from Journey log (h)	34 ± 14	20	28	58	4
Hours in transit (h)	24 ± 4	18	25	31	4
Load weight from CMR (kg)	12,164.00 ± 775.55	11,200	12,500	13,020	15
Load weight from TRACES (kg)	11,098.82 ± 1097.25	9000	11,000	13,250	3

**Table 8:** Descriptive statistics for the variables related to driver’s information and journey conditions.

Variables	Mean ± S.D.	Min	Median	Max
Driver’s age (years)	49 ± 5	33	48	59
Driver’s experience (years)	19 ± 7	12	15	37
Total vehicle dimension (m^2^)	33.63 ± 0.56	33	33.75	34.8
Space allowance (m^2^/animal)	1.71 ± 0.11	1.57	1.69	1.93
Stocking density (kg/m²)	333.23 ± 31.95	266.30	331.90	382.90
Drinkers (*n*)	15.50 ± 6.83	4	19	21
Horses per vehicle (*n*)	19.75 ± 2.03	18	20	21

**Table 9:** Frequency table of journeys (*n* = 20) and unbroken horses (*n* = 395) for the categorical variables related to the vehicle and journey characteristics.

Variables	Journeys Count (%)	Horses Count (%)
Ramp flooring:		
Non-slip knurled metal with foot battens	11/20 (55%)	219/395 (55%)
Corrugated metal	3/20 (15%)	119/395 (30%)
Rubber mat	6/20 (30%)	57/395 (15%)
Ramp side gates:		
No	5/20 (25%)	97/395 (25%)
Yes	15/20 (75%)	298/395 (75%)
Ramp covering:		
None	1/20 (5%)	19/395 (5%)
Partial	1/20 (5%)	20/395 (5%)
Complete	18/20 (90%)	356/395 (90%)
Vehicle bedding:		
Partial	4/20 (20%)	81/395 (21%)
Complete	16/20 (80%)	314/395 (79%)
Vehicle bedding type:		
Straw	11/15 (73%)	216/295 (73%)
Shavings	2/15 (13%)	38/295 (13%)
Straw and shavings	1/15 (7%)	21/295 (7%)
Sand and shavings	1/15 (7%)	20/295 (7%)
Group size:		
Three horses	15/19 (79%)	294/375 (78%)
Four horses	4/19 (21%)	81/375 (22%)
Adjacent stalls:		
No	14/20 (70%)	276/395 (70%)
Yes	6/20 (30%)	119/395 (30%)
Type of drinkers:		
Bowls	7/20 (35%)	138/395 (35%)
Nipples	13/20 (65%)	257/395 (65%)
Water tank:		
Full	4/20 (20%)	81/395 (20%)
Partially Empty	14/20 (70%)	276/395 (70%)
Empty	2/20 (10%)	38/395 (10%)
Space allowance classes:		
<1.75 m^2^/animal	15/20 (75%)	302/395 (76%)
>1.75 m^2^/animal	5/20 (25%)	93/395 (24%)
Fed during transport:		
Yes	9/15 (60%)	178/296 (60%)
No	6/15 (40%)	118/296 (40%)
Long stops (>12 h):		
No	11/19 (58%)	217/376 (58%)
Yes	8/19 (42%)	159/376 (42%)
Number of short stops:		
1	1/16 (6.25%)	19/315 (6.03%)
2	14/16 (87.50%)	276/315 (87.62%)
3	1/16 (6.25%)	20/315 (6.35%)
Season:		
Spring	6/20 (30%)	118/395 (30%)
Summer	1/20 (5%)	20/395 (5%)
Autumn	6/20 (30%)	115/395 (29%)
Winter	7/20 (35%)	142/395 (36%)

**Table 10:** Prevalence of health parameters observed within 30 min after unloading for the unbroken horses (*n* = 395) transported to the slaughterhouse in Southern Italy. The health issues are reported as the number of journeys with animals displaying the health issue on the 20 journeys and as the number of horses displaying the clinical sign on the 395 horses.

Health Parameters within 30 min after Unloading	N Journeys (Prevalence)	N Horses (Prevalence)
Injuries	6/20 (30%)	6/395 (1.52%)
Nasal discharge	7/20 (35%)	17/395 (4.30%)
Other discharges	6/20 (30%)	10/395 (2.53%)
Diarrhea and abnormal feces	10/20 (50%)	26/395 (6.58%)
Lameness	4/20 (20%)	7/395 (1.77%)

**Table 11:** Prevalence of health parameters 24 h after unloading for the unbroken horses (*n* = 395) and 20 journeys to a slaughterhouse in Southern Italy.

Health Parameters 24 h after Unloading	N Journeys (Prevalence)	N Horses (Prevalence)
Injuries	7/20 (35%)	7/395 (1.77%)
Nasal discharge	9/20 (45%)	19/395 (4.81%)
Other discharges	6/20 (30%)	9/395 (2.28%)
Diarrhea and abnormal feces	11/20 (55%)	27/395 (6.84%)
Lameness	4/20 (20%)	7/395 (1.77%)

**Table 12:** Significant associations between the predictive variables and the presence/absence of diarrhea and abnormal feces at 24 h after unloading. Data are presented as estimate, standard error (S.E.), odds ratio (OR), confidence interval (95% CI), and *p*-value.

Predictive Variable	Estimate ± S.E.	OR (95% CI)	*p*-Value
Arrival temperature	−0.160 ± 0.067	0.85 (0.73–0.96)	**0.017**
Season:			**0.003**
Autumn	Ref		
Winter	1.811 ± 0.633	6.12 (2.03–26.47)	0.004
Spring	-	-	n.s.
Summer	-	-	n.s.
Fed during transport:			**0.039**
Yes	Ref		
No	0.921 ± 0.445	2.51 (1.06–6.23)	0.038
Driver’s age	−0.247 ± 0.121	0.78 (0.61–0.98)	**0.041**
Stocking density	−0.067 ± 0.018	0.94 (0.90–0.97)	**0.0003**
Space allowance classes:			0.071 *
<1.75 m^2^/horse	Ref		
>1.75 m^2^/horse	0.764 ± 0.422	2.15 (0.91–4.85)	0.070

Ref: reference class; n.s.: not significant; * trend towards significance. Wald test *p*-values are in bold

**Table 13:** Final multiple regression model for the dummy dependent variable of presence/absence of diarrhea and abnormal feces 24 h after unloading. Data are presented as odds ratio (OR), confidence interval (95% CI), and *p*-value.

Predictive Variable	OR (95% CI)	*p*-Value
Season:		**<0.001**
Autumn	Ref	
Winter	5.72 (1.88–24.83)	0.006
Summer	-	n.s.
Spring	-	n.s.
Space allowance classes:		**0.120**
<1.75 m^2^/horse	Ref	
>1.75 m^2^/horse	1.94 (0.80–4.53)	0.128

Ref: reference class; n.s.: not significant. Wald test *p*-values are in bold.

**Table 14:** Significant associations between the predictive variables and the presence/absence of nasal discharge 24 h after unloading. Data are presented as estimate, standard error (S.E.), odds ratio (OR), confidence interval (95% CI), and *p*-value.

Predictive Variable	Estimate ± S.E.	OR (95% CI)	*p*-Value
Arrival temperature	−0.096 ± 0.070	0.91 (0.78–1.03)	**0.169**
Season:			**0.002**
Autumn	Ref		
Winter	2.441 ± 1.046	11.49 (2.24–210.32)	0.019
Spring	-	-	n.s.
Summer	-	-	n.s.
Long stops (>12 h):			**0.066**
Yes	Ref		
No	1.057 ± 0.573	2.88 (1.02–10.25)	0.065
Fed during transport:			**0.004**
Yes	Ref		
No	1.684 ± 0.585	5.38 (1.85–19.51)	0.004
Driver’s experience	−0.381 ± 0.179	0.68 (0.45–0.92)	**0.034**
Space allowance classes:			**0.169**
<1.75 m^2^/horse	Ref		
>1.75 m^2^/horse	0.676 ± 0.491	1.97 (0.71–5.05)	0.169

Ref: reference class; n.s.: not significant. Wald test *p*-values are in bold.

**Table 15:** Final multiple regression model for the dummy dependent variable of presence/absence of nasal discharge 24 h after unloading. Data are presented as estimate, standard error (S.E.), odds ratio (OR), confidence interval (95% CI), and *p*-value.

Predictive Variable	Estimate ± S.E.	OR (95% CI)	*p*-Value
Fed during transport:			**<0.001**
Yes	Ref		
No	1.754 ± 0.640	5.78 (1.83–23.91)	0.006
Arrival temperature	−0.731 ± 0.301	0.11 (0.02–0.42)	**0.002**
Space allowance classes:			**0.071**
>1.75 m^2^/horse	Ref		
<1.75 m^2^/horse	1.053 ± 0.702	2.86 (0.77–12.71)	0.018

Wald test *p*-values are in bold.

## Data Availability

The data presented in this study are available on request from the corresponding author.

## Supplementary Materials

The following supporting information can be downloaded at: https://www.mdpi.com/article/10.3390/ani12162083/s1, Table S1. Description of the measures considered in the protocol and collected during unloading (modified from Messori et al. [9]). Table S2. Definition of the measures collected on individual horses within 30 min of the unloading of the last animal and 24 h after unloading on the same horse in the lairage pens/stabling boxes (modified from Messori et al. [9]). Table S3. Measures collected on the vehicles after unloading the horses (modified from Messori et al. [9]). Table S4. Description of the Broken/Unbroken Test (BUT) developed by Menchetti et al. [18] and used to evaluate whether the transported horses were broken or unbroken the day after arrival. Table S5. Associations between the predictive variables and the presence/absence of abnormal feces 24 h after unloading in 395 horses transported to a slaughterhouse in Southern Italy. Associations are presented with Wald test *p*-values. Table S6. Associations between the predictive variables and the presence/absence of nasal discharge 24 h after unloading in 395 horses transported to a slaughterhouse in Southern Italy. Associations are presented with Wald test *p*-values. Video S1. Self-unloading recorded upon arrival of one of the vehicles assessed in the study. Video S2. An example of a BUT test performed with one of the horses assessed in this study.
