# Supplementary material for: Journeys, Journey Conditions, and Welfare Assessment of Unbroken (Unhandled) Horses on Arrival at a Slaughterhouse in Italy
Source: Animals (Basel). 2022 Aug 15;12(16):2083. doi: 10.3390/ani12162083 (PMC9404719; doi:10.3390/ani12162083)
Supplement: Supplementary file 1 [file animals-12-02083-s001.zip › Supplementary Zappaterra unbroken horses submitted.pdf]

Supplementary material

Table S1. Description of the measures considered in the protocol and collected during unloading (modified from Messori et al, 2016 [9]).

Measure	Description
Duration of unloading	Record the time from the lowering of the vehicle ramp until the last horse was unloaded.
Total animals transported	Record the number of animals transported
Dead on arrival (DOA)	Record the number of animals found dead in the vehicle on arrival.
Non-ambulatory*	Record the number of animals that cannot rise or are unable to stand or walk unaided.
Reluctant to move*	Record the number of animals per vehicle that are showing unwillingness to go forward (not caused by physical problems) or suddenly stopping during the unloading for at least 3 s.
Severely lame*	Record the number of animals per vehicle that are showing reduced ability to use one or more limbs in a normal manner. Score only severe lameness (i.e., reluctance to bear weight on one foot).
Slipping/losing balance**	Record the number of animals per vehicle that are showing a loss of balance during unloading, without a non-limbic part of the body touching the ground.
Falling**	Record the number of animals per vehicle that are showing a loss of balance during unloading causing any part of the body (other than hooves) to touch the ground.
Galloping or jumping	Record the number of animals per vehicle that gallop or jump approaching the ramp, on the ramp and/or within 3 m after the point where the ramp touches the ground.
Body Condition Score	Record the number of animals per vehicle that are presenting poor (0–1) or too high (5) BCS, according to the scoring technique by Carrol and Huntington (1988).
Sweating	Record the number of animals per vehicle that are presenting visible signs of sweating (i.e., wet animals, dried sweat spots, salt deposits).
Severe injuries	Record the number of animals per vehicle that have wounds through the skin which involve damage to deeper tissue (i.e., muscles, tendons), a cut through the skin so big that it would normally be stitched or with extensive and serious injuries that may cause loss of function over a long period of time (e.g., serious damage to a tendon or joint, fracture). Scoring technique for severe injuries adapted from Jørgensen et al (2009).
Exhaustion	Record the number of animals showing severe fatigue or exhaustion (e.g., chin or limbs resting at partitions or troughs, closed eyes, high drive to rest in recumbent position).
Coughing	Record whether it is possible to hear more than one cough

Appropriate handling***	Assess whether or not the handler gives the horses the opportunity to slow down and inspect the ramp and if the handler is able to control the speed of movement.
The handler moves the animals in an arousing manner, inducing fear ***	Assess whether or not the handler is moving the animals in an arousing manner (i.e., rapid movements of arms or body that could induce fear reactions in the animals).
The handler moves the animals in a noisy manner, inducing fear ***	Assess whether or not the handler is making loud noises to induce the animals to move, potentially inducing fear reactions (i.e., banging on the vehicle wall/metal partitions, shouting).
The handler hits the animals without reason ***	Assess whether or not the handler is slapping the animal without reasons (e.g., the animal is already moving) or to make them move when they are not in the condition to do it.
The handler moves the animals properly	This record is mutually exclusive with the previous ones. The handler moves the animals properly when he does not induce fear, nor performs prohibited practices
The handler performs prohibited practices ***	Assess whether or not the handler is performing forbidden practices, according to Regulation EC 1/2005:<br>• strike or kick the animals;<br>• press sensible areas to cause unnecessary pain;<br>• suspend the animals by mechanical means;<br>• lift or drag the animals by the head, ears, legs or tail or handle them in such a way as to cause them unnecessary pain or suffering;<br>• use prods or other implements with pointed ends; and<br>• knowingly obstruct any animal which is being driven or led through any part where animals are handled.

* "Non-ambulatory", "reluctance to move" and "severe lameness" are mutually exclusive.
** "Slipping" and "falling" are mutually exclusive.
*** Measures assessed at the vehicle level.

Table S2. Definition of the measures collected on individual horses within 30 minutes of the unloading of the last animal and 24 h after unloading on the same horse in the lairage pens/stabling boxes (modified from Messori et al, 2016 [9]).

Measure	Description
Sex	Record the gender of the horses
Halter	Record whether the horse wears a halter
Horse type	Record if a horse is draft or light type.
Body Condition Score (BCS)	Record the BCS of each horse, according to the scoring technique by Carrol and Huntington (1988).
Estimated weight	Estimate the body weight of the horses by visual inspection and experience
Demeanor*	Assessed through qualitative behaviour assessment set on a limited observation period in which the assessor focuses on 'how' the animal expresses any given behaviour (Dalla Costa et al 2014). According to that, descriptors are fixed in order to use the following same subjective terms:

	Alert, responsive, awake: vigilant, more responsive to external stimuli, vocalize and interacts a lot;<br>Alert, responsive, calm: vigilant, quite responsive to external stimuli, few interactions;<br>Depressed: disengaged from and unresponsive to the environment, quiet, apathetic;<br>Exhausted: tired, eyes shut, does not interact, may lay down or be motionless<br>Terrified: restless, vocalizes a lot.
Heart rate	Pulse rate assessed at the level of facial artery
Respiratory rate	Assessed by looking at the abdomen/flank area of the horse to look at the rise and fall. Count the number of breaths for 15 seconds and multiply the amount by four to calculate the breaths per minute.
Coat	Assess if the horse has long or short coat
Sweating	Assess if the horse has visible signs of sweating (i.e., wet animals, dried sweat spots, salt deposits)
Feces*	Record the type of feces (i.e., not formed, normal, diarrhea).
Clinical signs of colic	Record the number of animals showing signs of abdominal discomfort (e.g., entero-colitis).
Tail*	Assess the presence/absence of damaged tail and the entity of the damage (i.e., no damage, ruffled tail from rubbing, open skin lesion).
Nasal Discharge*	Assess the presence/absence of unilateral or bilateral nasal discharge
Type of nasal discharge	Record the type of secretion produced (i.e., watery, purulent, bloody mucus, smelly).
Other discharges*	Assess the presence/absence of discharge in other parts (i.e., eyes, mouth, rectum, vulva, penis).
Cough	Record the presence of coughing events. Coughing is defined as a sudden and noisy expulsion of air from the lungs. Record if more or less than one coughing events occur during the whole observation in lairage pens.
Cuts or injuries	Assess whether the horse has wounds through the skin, a cut through the skin so big that it would normally be stitched or with extensive and serious injuries that may cause loss of function over a long period of time.
Lameness score	Lameness was scored as follows: 0=sound; 1=lameness difficult to detect, apparent intermittent claudication; 2=lameness in some cases difficult to detect, which becomes more evident in certain circumstances; 3=lameness observable at trot; 4=lameness with head nodding; 5=minimal weight bearing and inability to move.
Other clinical signs	Report other possible health issues.

* Additional measures not present in Messori et al (2016).

Table S3. Measures collected on the vehicles after unloading the horses (modified from Messori et al, 2016 [9]).

Measure	Description
Vehicle covering	Record the presence of a tarpaulin over the outside of the vehicle, entirely covering one of the lateral sides.
Forced ventilation*	Indicate the number of ventilators and if they are functioning.
Cameras*	Indicate the number of cameras and if they are functioning.
Horses in group	Indicate the number and the dimensions of the stall inside the vehicle.
If tied	If the horses are tied inside the vehicle, report the rope length and if it is appropriate to allow head movements (at least 60 cm).
Deck height	Indicate if the height of the deck is adequate for the transported animals (i.e., 75 cm higher than the withers of the highest animal).
Ramp height	Record the height (H) of the ramp 1 m from the terminal projection of the ramp from which the slope can be calculated. If H is more than 36.4 cm, it is non-compliant with the requirements of the Regulation.
Direction of travel	Record the direction of the horses inside the vehicle in relation to the direction of the travel (90°, 45°, facing forward or backward).
Adjacent stalls	Record the presence of any stallion adjacent to a mare or to another stallion in the vehicle.
Ramp flooring type	Indicate the type of ramp flooring (i.e., smooth metal, corrugated metal, rubber mat).
Ramp flooring conditions	Indicate if the ramp floor is adequate (i.e., intact, not presenting harmful surfaces or slippery areas and if it is well-drained).
Ramp covering	Ramp covering prevents light reflection, deadens sounds and reduces the slippery areas. Indicate the type of bedding on the ramp (i.e., straw, wood-shaving, sand) and the quantity (i.e., ramp totally covered/partially covered so that it is possible to see the ramp surface/none).
Ramp lateral protections	Indicate if the ramp lateral protections are present and if they present openings or sharp edges.
Slots/Steps	Steps and gaps are any height difference or hollow space of more than 10 cm between two surfaces. Indicate the presence of any gap or step potentially endangering or frightening the animals, being between lorry/ramp/floor or lorry door/lateral protection.
Ramp slope	Measure the ramp slope, if it is more than 20°, it is non-compliant with the requirements of the Regulation
Blockage zones on the ramp	Indicate the presence of any blocking zone (i.e., shadows, holes or physical obstacles) on the ramp.

Lighting for animal orientation	Indicate the presence of adequate lighting (i.e., sufficient to allow the animals to orientate, and that is not reflecting on the ramp or directly orientated toward the unloading animals.
Lighting for animal movement during unloading	Indicate the presence of adequate lighting (i.e., allowing the assessor to read the scoring sheet) at unloading for handling purposes.
Sharp edges	Indicate the presence/absence of sharp or harmful edges inside the vehicle.
Number of partitions	Record the number of partitions/compartments
Dimensions of bays/compartments	Records the dimensions of bays/compartments (width and length)
Dimensions of the vehicle (m²)	Record the dimensions (width and length) of the vehicle and calculate the total space (m²)
Space allowance	Calculate the space allowance per animals in the vehicle (i.e., total deck area minus projection of the area of partitions on the floor/number of horses).
Vehicle floor covering	Bedding provides a more comfortable resting surface; it helps absorb urine and feces and reduces the risk of slipping. Indicate the type of bedding on the vehicle (i.e., straw, wood-shaving, sand) and its quantity (i.e., totally covers the deck/partially covers the deck so that it is possible to see the deck surface/none).
Drinkers	Indicate the number and type (i.e., nipples, bowls) of drinkers and if they are functioning.
Water tank	Record the amount of water on the vehicle tank (full/empty/nor full nor empty).
Temperature monitoring and control systems	Record the presence of the temperature monitoring and control system in the vehicle and if it is functioning.
Signs of diarrhea in the vehicle	Indicate if there are signs of diarrhea inside the vehicle.
Documents/information checklist	Assess the presence of horses' passports, International Consignment note, TRACES, Journey Log and the weight of the load.

* Additional measures, not present in Messori et al. (2016).

Table S4. Description of the Broken/Unbroken Test (BUT) developed by Menchetti et al (2021) [18] and used to evaluate whether the transported horses were broken or unbroken the day after arrival.

Phase	Description
Broken/Unbroken test	Always performed by the same tester, consist in two phases Approaching/Haltering test and Handling test which are used to evaluate horse behavior during approach, haltering and leading. Based on the tolerance test proposed by Wulf et al., this test must have a maximum duration of 10 minutes. At first signs of pain or high level of distress the procedure is stopped. At the end, the tester judges if the horse is broken or unbroken based on the validated score.
Approach / Halter test	, the tester enters the test area and walks toward the horse slowly with the halter in hand, approaching and then trying to halter the horse. Maximum time allowed for this test is 5 minutes. After that, if it was possible to halter the horse, the tester would start the Handling test.

Handling test	the tester tries to lead the horse three steps forward and three steps backwards. When leading, the handler keeps the horse relatively close to her right-hand side. The tester uses a negative reinforcement procedure, applying light pressure on the lead rope and releasing the pressure as soon as the horse shows the desired behavior (e.g., a step forward). Maximum time allowed for this test is 5 minutes.

Table S5. Associations between the predictive variables and the presence/absence of abnormal feces 24 h after unloading in 395 horses transported to a slaughterhouse in Southern Italy. Associations are presented with Wald Test P-values.

Variable	Wald test P-value
Departure country	0.658
Country of origin	0.112
Duration from TRACES	0.103
Duration from Journey Log	0.809
Hours in transit	0.332
Unloading duration	0.345
Driver's age	0.042
Driver's experience	0.123
Arrival temperature	0.017
Season	0.003
Long stops (>12h)	0.292
Number of short stops	0.998
Fed during transport	0.039
Group size	0.988
Adjacent bays	0.104
Ramp bedding	0.450
Ramp covering	0.279
Ramp lateral protection	0.096
Space allowance classes	0.071
Stocking density	0.0003
Vehicle bedding	0.001
Type of drinkers	0.057
Nasal discharge	0.987

Table S6 Associations between the predictive variables and the presence/absence of nasal discharge 24 h after unloading in 395 horses transported to a slaughterhouse in Southern Italy. Associations are presented with Wald Test P-values.

Variable	Wald test P-value
Departure country	0.963
Country of origin	0.661
Duration from TRACES	0.327
Duration from Journey Log	0.104
Hours in transit	0.285
Unloading duration	0.175
Driver's age	0.161
Driver's experience	0.034
Arrival temperature	0.169
Season	0.002
Long stops (>12h)	0.066
Number of short stops	0.841
Fed during transport	0.004
Group size	0.988
Adjacent bays	0.382
Ramp bedding	0.994
Ramp covering	0.005
Ramp lateral protection	0.006

Space allowance classes	0.169
Stocking density	0.502
Vehicle bedding	0.005
Type of drinkers	0.999
Abnormal feces	0.990

Video S1 Self-unloading recorded upon arrival of one the vehicle assessed in the study

Video S2 An example of a BUT test performed with one of the horses assessed in this study
